# *In vitro* and *in vivo* antifungal activities and mechanism of heteropolytungstates against *Candida* species

**DOI:** 10.1038/s41598-017-17239-8

**Published:** 2017-12-05

**Authors:** Han Li, Hongwei Gong, Yanfei Qi, Juan Li, Xufeng Ji, Jiaheng Sun, Rui Tian, Hao Bao, Xiangfu Song, Qiang Chen, Guoliang Liu

**Affiliations:** 1 School of Public Health, Jilin University, Changchun, Jilin, 130021 P. R. China; 2 Department of Infection Control, The First Hospital of Jilin University, Changchun, Jilin, 130021 P. R. China; 3 Department of Laboratory Medicine, The First Hospital of Jilin University, Changchun, Jilin, 130021 P. R. China

## Abstract

The antifungal activities of heteropolytungstates, α-1,2,3-K_6_H[SiW_9_V_3_O_40_] (SiW-3), K_13_[Ce(SiW_11_O_39_)_2_]·17H_2_O (SiW-5), K_13_[Eu(SiW_11_O_39_)_2_]·25H_2_O (SiW-10), K_6_PV_3_W_9_O_40_ (PW-6), α-K_4_PVW_11_O_40_ (PW-8), were screened in 29 *Candida albicans*, 8 *Candida glabrata*, 3 *Candida krusei*, 2 *Candida parapsilosis*, 1 *Candida tropicalis*, and 1 *Cryptococcus neoformans* strains using the CLSI M27-A3 method. SiW-5 had the highest efficacy with a minimum inhibitory concentration (MIC) values of <0.2–10.2 *μ*M *in vitro*. The antifungal mechanism, acute toxicity and *in vivo* antifungal activity of SiW-5 were then evaluated in *C. albicans*. The results showed that SiW-5 damaged the fungal cell membrane, reduce the ergosterol content and its main mode of action was through inhibition of ergosterol biosynthesis. Real-time PCR showed that ERG1, ERG7, ERG11 and ERG28 were all significantly upregulated by SiW-5. An acute toxicity study showed the 50% lethal dose (LD_50_) of SiW-5 for ICR mice was 1651.5 mg/kg. And *in vivo* antifungal studies demonstrated that SiW-5 reduced both the morbidity and fungal burden of mice infected with *C. albicans*. This study demonstrates that SiW-5 is a potential antifungal candidate against the *Candida* species.

## Introduction

Following the ever increasing application of antibiotics, immunosuppressive agents, and invasive medical devices, as well as increasing numbers of immunocompromised patients, fungal infections have dramatically increased worldwide^1,2^. Among fungal infections, the *Candida* species is one of the dominant fungal pathogens, associated with high rates of morbidity and mortality^2,3^. However, the development of antifungal drugs has always lagged behind fungal infections incidence, and most of antifungals have limited potential as systemic agents due to issues of toxicity, adverse effects or restricted bioavailability. Moreover, their historic long-term application has resulted in many drug resistance^4,5^. Therefore, there is an increasing need for novel, more efficient and safer antifungals.

Polyoxometalates (POMs) are early transitional metal oxygen anion clusters and have garnered worldwide attention due to the versatility of their chemical structures. They have numerous applications in chemistry, materials science, catalysis, redox, magnetism, medicine, and have potential to be especially promising as antibacterial, antiviral and antitumor agents^6–8^. To the best of our knowledge, there are only a small number of reports on their antibacterial activities^9–11^, and no reports on their antifungal activities.

Herein, a series of antifungal susceptibility tests were carried out for heteropolytungstates, α-1,2,3-K_6_H[SiW_9_V_3_O_40_] (SiW-3), K_13_[Ce(SiW_11_O_39_)_2_]·17H_2_O (SiW-5), K_13_[Eu(SiW_11_O_39_)2]·25H_2_O (SiW-10), K_6_PV_3_W_9_O_40_ (PW-6), and α-K_4_PVW_11_O_40_ (PW-8). To investigat the antifungal mechanism of heteropolytungstates, the ultrastructure of *C. albicans* was visualized by transmission electron microscopy (TEM), the ergosterol contents were measured by high performance liquid chromatography (HPLC), and real-time PCR was carried out to evaluate the inhibition of heteropolytungstates on the ergosterol biosynthesis at the molecular level. In addition, the *in vivo* efficacy of heteropolytungstate in a *C. albicans* systemic infection murine model was evaluated.

## Results

### Characterization of Compounds

The compounds were prepared according to the literature and identified by FI-IR spectrum, UV-Vis spectrum, as shown in Fig. S1 and Table S1, and Fig. S2. These bands in the spectra correspond to those found in the literature^12–16^. The representation of structures of heteropolytungstates are showed in Fig. S3.

### MIC determination

Table 1 showed the MICs of heteropolytungstates against fungal strains. SiW-3, PW-6, PW-8 and SiW-10 exhibited the antifungal activity with the MICs of 3.0 - > 188.9, 0.7 - > 188.8, 0.7 - > 176.6, and 0.2 - > 79.3 *μ*M respectively. SiW-5 had the highest efficacy with MIC range of < 0.2–10.2 *μ*M, which was higher than that (<0.4–209.2 *μ*M) of FLC. Therefore, we chose SiW-5 for further studies against *C.albicans* HL27.

**Table 1:** MIC values (*μ*M) of FLC and heteropolytungstates against fungi.

Strains	FLC	SiW-5	SiW-10	SiW-3	PW-6	PW-8
***C. albicans***						
HL 24	0.8	5.1	5.0	94.5	188.8	>176.6
HL 25	0.8	5.1	5.0	188.9	188.8	>176.6
HL 26	0.4	2.5	2.5	>188.9	>188.8	>176.6
HL 27	0.8	0.6	2.5	188.9	188.8	>176.6
HL 28	0.4	1.3	19.8	>188.9	>188.8	>176.6
HL 29	0.4	2.5	19.8	94.5	94.4	>176.6
HL 32	52.3	1.3	2.5	5.9	11.8	11.0
HL 33	26.1	0.6	1.2	11.8	2.9	1.4
HL 34	13.1	0.6	1.2	5.9	5.9	1.4
HL 35	13.1	1.3	2.5	5.9	2.9	2.8
HL 36	6.5	1.3	0.6	11.8	11.8	5.5
HL 37	6.5	1.3	0.6	3.0	5.9	2.8
ATCC 90028	0.4	5.1	39.6	>188.9	>188.8	>176.6
HL 59	0.4	1.3	2.5	23.6	94.4	88.3
ATCC 14053	0.8	5.1	19.8	>188.9	>188.8	>176.6
HL 65	1.6	2.5	5.0	188.9	188.8	>176.6
HL 66	0.4	1.3	0.6	188.9	188.8	>176.6
HL 67	0.8	10.2	>79.3	>188.9	>188.8	>176.6
HL 68	0.4	5.1	19.8	>188.9	>188.8	>176.6
HL 72	209.2	1.3	1.2	47.2	94.4	>176.6
HL 73	<0.4	1.3	2.5	188.9	188.8	>176.6
HL 74	0.8	5.1	9.9	>188.9	>188.8	>176.6
HL 75	<0.4	2.5	2.5	188.9	188.8	>176.6
HL 76	0.8	2.5	2.5	188.9	188.8	>176.6
HL 77	0.4	2.5	2.5	188.9	188.8	>176.6
HL 78	0.4	2.5	9.9	188.9	188.8	>176.6
HL 79	0.4	2.5	5.0	>188.9	>188.8	>176.6
HL 80	0.8	5.1	9.9	188.9	>188.8	>176.6
HL 81	<0.4	2.5	5.0	188.9	>188.8	>176.6
***C. glabrata***						
ATCC 90030	26.1	2.5	9.9	94.5	47.2	22.1
HL 62	3.3	0.3	0.3	23.6	47.2	5.5
HL 69	3.3	0.3	0.6	23.6	11.8	2.8
HL 70	13.1	1.3	5.0	188.9	94.4	>176.6
HL 71	6.5	<0.2	0.2	11.8	2.9	0.7
HL 83	0.8	2.5	79.3	>188.9	>188.8	>176.6
HL 84	13.1	0.3	0.3	5.9	2.9	1.4
HL 85	<0.4	1.3	2.5	94.5	1.5	11.0
***C. krusei***						
HL 31	6.5	0.3	0.3	5.9	0.7	1.4
ATCC 6258	52.3	1.3	2.5	23.6	47.2	>176.6
HL 61	26.1	2.5	9.9	>188.9	11.8	44.1
***C. parapsilosis***						
ATCC 22019	3.3	5.1	5.0	47.2	47.2	22.1
HL 63	3.3	2.5	5.0	94.5	94.4	5.5
***C. tropicalis***						
HL 60	0.4	1.3	9.9	47.2	47.2	22.1
***C. neoformans***						
HL 30	6.5	5.1	2.5	11.8	11.8	11.0

MIC values were determined according to CLSI protocol M27-A3 (2008) using prominent decrease in turbidity as the cutoff for the reported MIC values. FLC, fluconazole.

### Time kill kinetics

**Figure 1:**
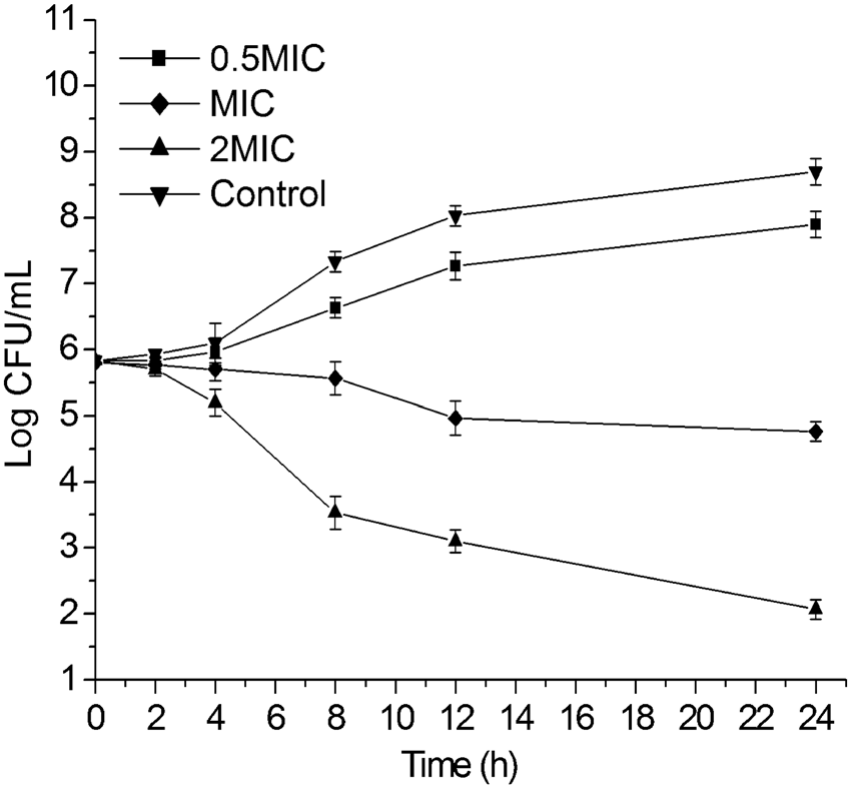
Time kill curve for *C. albicans* HL27 treated with 0.5 MIC (0.3 *μ*M), MIC (0.6 *μ*M) and 2 MIC (1.2 *μ*M) of heteropolytungstate SiW-5. The experiment was performed in triplicate. Data were represented as mean ± SD.

The antifungal activity of SiW-5 was confirmed by the time kill test. As shown in the Fig. 1, no appreciable antifungal activity of SiW-5 at 0.5MIC was observed. But the groups at MIC and 2MIC showed fungicidal effect and led to a decrease of 3.93 and 6.63- log_10_CFU/mL at 24 h. The inhibition of the SiW-5 continuously increased with the enhancement of the concentration and time, indicating that the antifungal effect of SiW-5 is time- and dosed dependent.

### Transmission electron microscopy (TEM)

**Figure 2:**
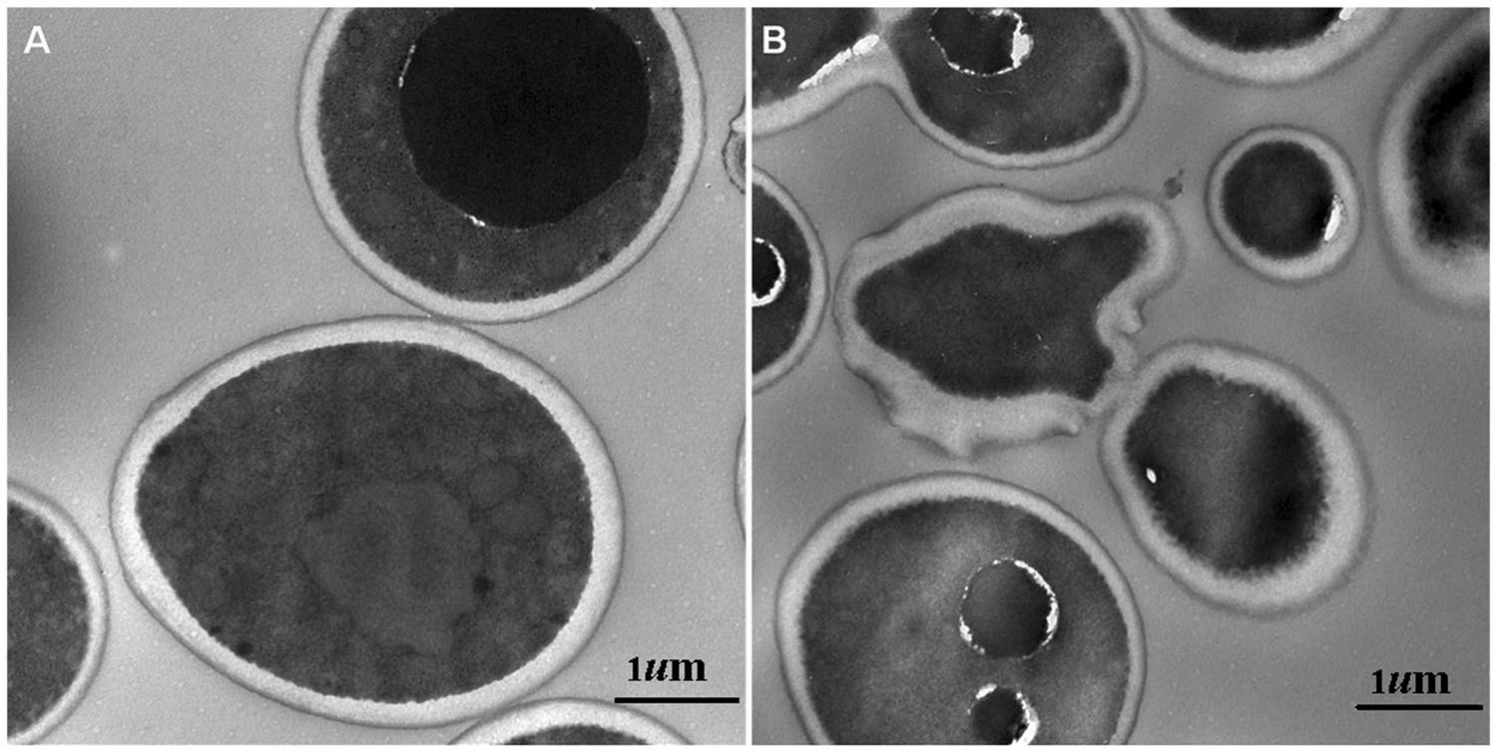
The effect of heteropolytungstate SiW-5 on the cell ultrastructure changes of *C. albicans* HL27 by transmission electron microscopy (×3k). Untreated cells (**A**) and cells treated with MIC value (0.6 *μ*M) of SiW-5 (**B**) were incubated for 24 h and stained with uranyl acetate and lead citrate. Scale bar: 1 *μ*m.

TEM was used to study the ultrastructure changes of the *C. albicans* HL27 treated by SiW-5. As shown in Fig. 2A, the *C. albicans* HL27 cells in control groups showed uniform density enveloped with an intact and regular cell wall and membrane. While treated with 0.6 *μ*M of SiW-5 for 24 h, the cells were smaller and irregular. The cell wall and membrane were rough and the intracellular content leakage. Moreover, structural disorganization in the cytoplasm were clearly observed, shown in Fig. 2B.

### Assessment of ergosterol content

**Figure 3:**
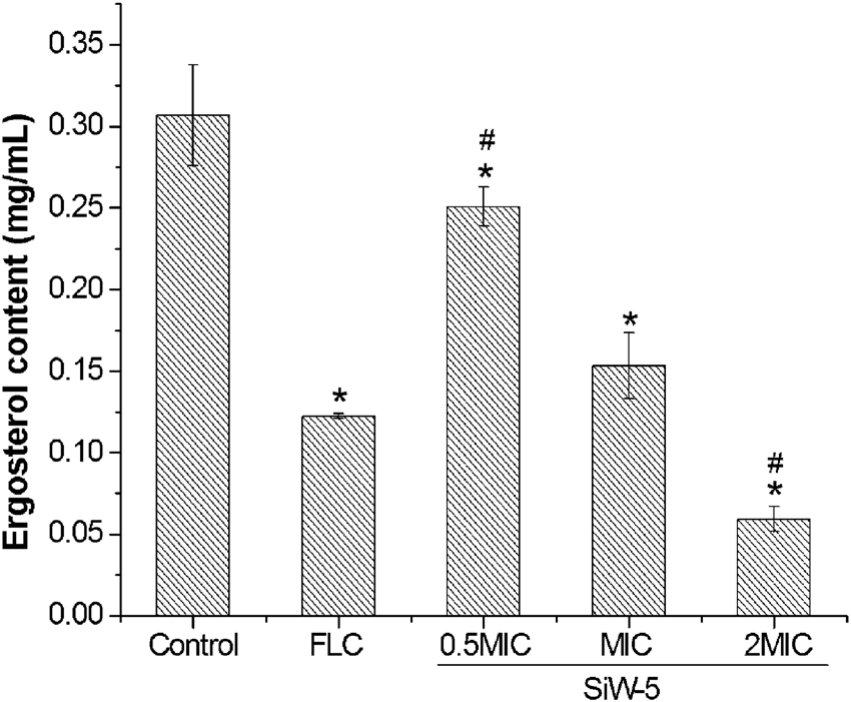
Concentration changes of ergosterol in *C. albicans* HL27 treated with 0.5 MIC (0.3 *μ*M), MIC (0.6 *μ*M) and 2 MIC (1.2 *μ*M) of heteropolytungstate SiW-5 and MIC (0.8 *μ*M) of FLC at 24 h using HPLC method. The experiment was performed in triplicate. Data were represented as mean ± SD. *P < 0.05 for the SiW-5 at the concentration of 0.5 MIC (0.3 *μ*M), MIC (0.6 *μ*M), 2 MIC (1.2 *μ*M) or FLC at MIC (0. 8 *μ*M) *vs*. control (untreated cells). ^#^ *P* < 0.05 for the SiW-5 at the concentration of 0.5 MIC (0.3 *μ*M) or 2 MIC (1.2 *μ*M) *vs*. FLC at MIC (0.8 *μ*M). FLC, fluconazole.

Ergosterol is an important constituent of fungal membrane for maintaining cell integrity, membrane fluidity and the cell metabolism. It is the target of most existing antifungals. Therefore, the effect of SiW-5 on the ergosterol content of *C. albicans* was conducted by HPLC. The HPLC results showed that the retention time of ergosterol was about 14.798 min. The standard curve was linear and *R* ^2^ = 0.9999 (Fig. S4). The ergosterol inhibition ratios of MIC of FLC and 0.5MIC, MIC and 2MIC of SiW-5 were 61.29%, 19.35%, 51.61%, and 80.65%, respectively (Fig. S5, Fig. 3). The inhibition ratio of SiW-5 on ergosterol synthesis continuously increased with the enhancement of concentration, indicating that the antifungal effect of SiW-5 was dependent on its concentrations and its main mode of action was through inhibition of ergosterol biosynthesis.

### Real-Time PCR

To study the interference of ergosterol biosynthesis caused by SiW-5 on a molecular level, real-time PCR was performed. The *C. albicans* HL27 cells were exposed to SiW-5 (MIC values) for 24 h, their total RNA was extracted, and cDNA synthesized. This cDNA was then used as a template for a series of RT-PCRs. The *ERG1*, *ERG7*, *ERG11* and *ERG28* genes were found significantly upregulated with the fold change relative to control in gene expression of 5.95 ± 1.30, 4.90 ± 0.24, 7.40 ± 2.56 and 6.29 ± 0.71, respectively. No obvious change was found in the expression of *ERG27* with the relative fold change of 1.47 ± 0.46.

### Acute toxicity

*In vivo* toxicity of SiW-5 was evaluated in healthy ICR mice. The LD_50_ in ICR mice was determined to be 1651.5 mg/kg by the Bliss method, and the confidence level of 95% was 1539.6 mg/kg to 1926.5 mg/kg, which was classified as low toxicity. There were no adverse effects or clinical signs of toxicity for the surviving mice, and no significant influence of SiW-5 on body weight during the observation period.

### *In vivo* antifungal studies

**Figure 4:**
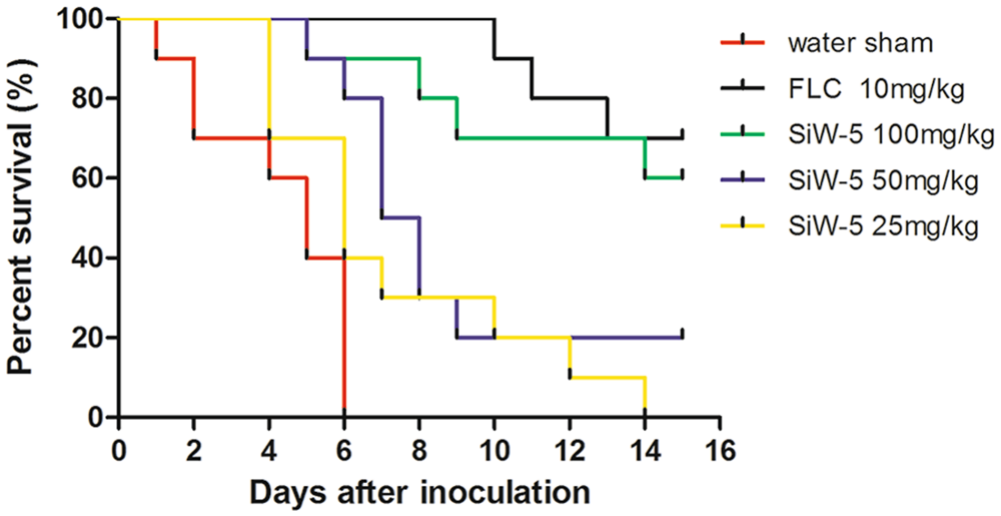
Survival rates of mice with systemic candidiasis. Immunosuppressed BALB/c mice were infected with 2 × 10^5^ CFU/mL of *C. albicans* HL27 in 0.1 mL sterile saline via tail vein, and were treated with sterile distilled water, 10 mg/kg FLC, 25, 50 or 100 mg/kg heteropolytungstate SiW-5 intraperitoneally (*P* < 0.05, Kaplan-Meier test). FLC, fluconazole.

**Figure 5:**
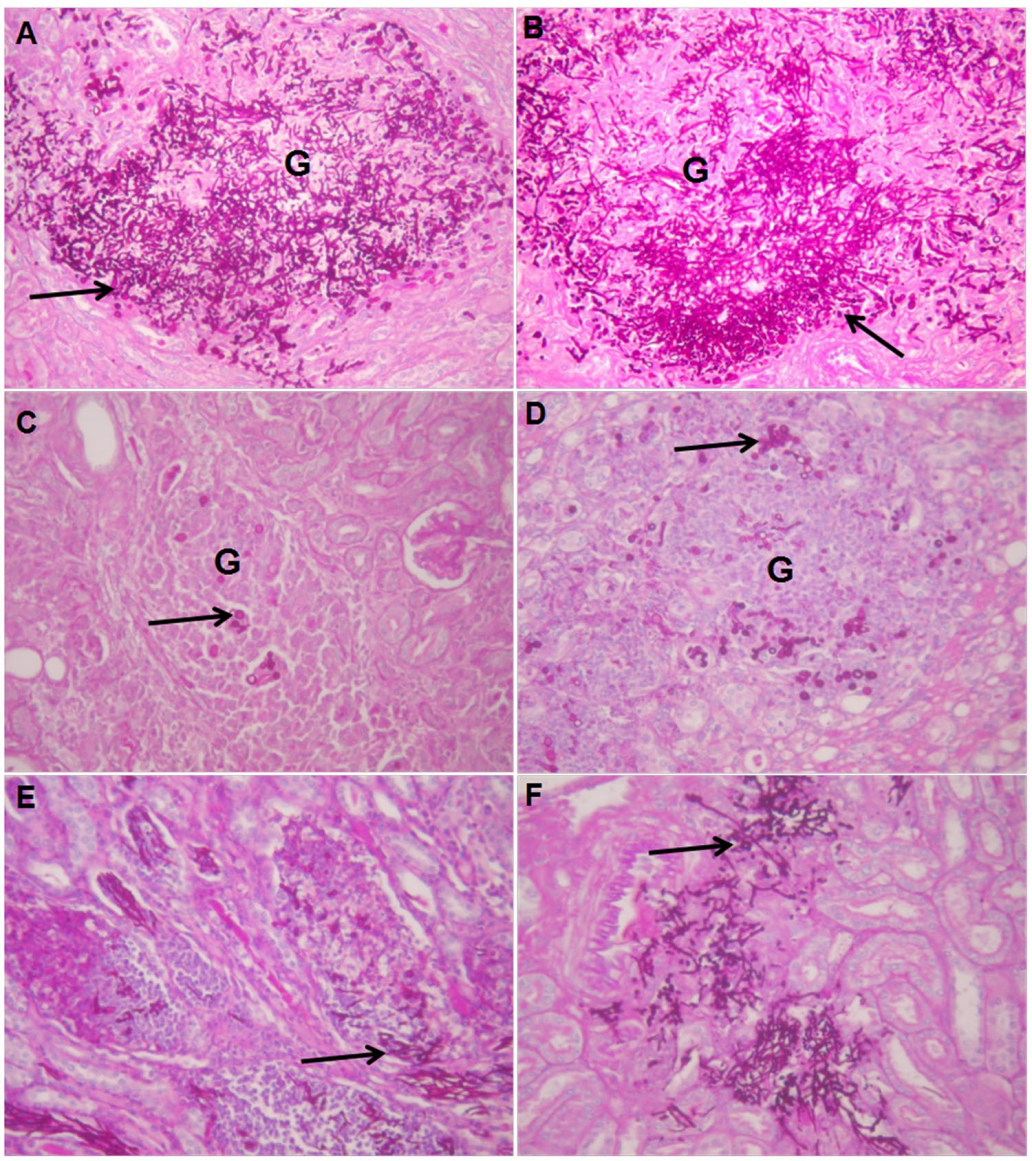
Representative PAS-stained histological features of kidneys from mice with systemic candidiasis (×200 magnification). Immunosuppressed BALB/c mice were infected with 2 × 10^5^ CFU/mL of *C. albicans* HL27 in 0.1 mL sterile saline via tail vein, and were treated with sterile distilled water (**A**), 10 mg/kg FLC (**B**), 25 mg/kg heteropolytungstate SiW-5 (**C**), 50 mg/kg SiW-5 (**D**) or 100 mg/kg SiW-5 (**E**) intraperitoneally. Arrow shows fungal yeasts and hyphae. (**G**) Represents typical granulomas. FLC, fluconazole.

The *in vivo* efficacy of SiW-5 or FLC was determined against a systemic infection by *C. albicans* HL27 in mice with CY-induced immunosuppression. The survival ratios over time of mice treated with SiW-5 and FLC were shown in Fig. 4. The water-sham group all died within 6 days, and the median survival time was 5 days. The groups treated with 10 mg/kg FLC and 100 mg/kg SiW-5 showed 70% and 60% survival after 15 days. The median survival time of the groups treated with 50 mg/kg and 25 mg/kg SiW-5 were 7 and 6 days. PAS-stained histological examinations of kidneys from infected mice treated with sterile water showed obvious tissue damage and a large amount of yeasts and hyphae, which slightly and moderately decreased in the group treated with 25 mg/kg and 50 mg/kg SiW-5. While in the groups treated with 100 mg/kg SiW-5 and 10 mg/kg FLC, the fungal cells were markedly reduced and showed a yeast form (Fig. 5). Treatments with 100, 50 and 25 mg/kg SiW-5 and 10 mg/kg FLC caused significant reduction in CFU counts per gram of kidney compared to the water sham group (Table 2).

**Table 2:** Kidney fungal burden of mice with systemic candidiasis.

Drugs	Dose (mg/kg)	log CFU/g in kidney^a^
Water sham	—	6.63 ± 0.06
FLC	10	3.26 ± 0.93^b^
SiW-5	25	5.51 ± 0.45^b^
SiW-5	50	4.82 ± 0.15^b^
SiW-5	100	3.91 ± 0.09^b^

^a^Immunosuppressed BALB/c mice were infected with 2 × 10^5^ CFU/mL of *C. albicans* HL27 in 0.1 mL sterile saline via tail vein, and were treated with sterile distilled water, 10 mg/kg FLC, 25, 50 or 100 mg/kg heteropolytungstate SiW-5 intraperitoneally. The kidney fungal burden was presented as log CFU per gram of kidney value. The experiment was performed in triplicate. Data were represented as mean ± SD. ^b^ *P* < 0.05 for FLC and SiW-5 *vs*. water sham. FLC, fluconazole.

## Discussion

*Candida* species are the main fungal pathogen that causes infections in humans, ranging from superficial mucosal infection to systemic mycoses^17^. *Candida* infections are recurrent diseases, which have increased due to the rise in the number of immunocompromised host populations^18^.

Sun *et al*. reported the antifungal activity of organothiophosphoryl polyoxotungstates against *Fusarium graminearum* in 2002^19^. Until now, antifungal activities of polyoxometalates against human fungal pathogens are seldom reported. In present study, the antifungal activities of a series of heteropolytungstates were evaluated through MIC determination compared with FLC. The test compounds exhibited potent antifungal activities against fungal strains. Among them, SiW-5 has the highest efficacy, and higher than that of the positive control group FLC. The structure of the polyoxotungstates seems to be important for the antifungal activity. The lacunary-Keggin sandwiched polyoxotungstates and Keggin-structural polyoxotungstates are known to exhibit various biological activities such as the anti-human immunodeficiency virus. Herein, the antifungal activity of lacunary-Keggin sandwiched polyoxotungstates is higher than the Keggin-structural polyoxotungstates. Maybe the stability of the lacunary-Keggin sandwiched polyoxotungstates is higher than the others at pH 7.5. The charge number of the polyoxotungstates is also an important factor. The most potent antifungal polyoxotungstates, SiW-5 and SiW-10, show highly negative charges of 13, while the others showing weak antifungal activities have lower negative charges. The polyoxotungstates, especially highly negative-charged lacunary-Keggin sandwiched polyoxotungstates, exhibited potent antifungal activities.

Based on the previous studies, the antiviral property of heteropolytungstate Cs_2_K_4_Na[SiW_9_Nb_3_O_40_]·H_2_O is mainly due to its localization on the host cell surface, inhibiting viral adsorption. In addition, polyoxotungstates with antibacterial activity were preferentially located on the membrane of bacteria with intact composition^20^. And the antibacterial polyoxotungstates uptaken in the cell were preferentially located on the membrane with intact composition^21^. Therefore, the fungal membrane maybe the target of SiW-5^22^. Ergosterol is an important component throughout the fungal cell membranes, which distinguishes fungi from bacteria, plant and animal cells. It plays a vital role in many biological functions such as maintaining cell integrity, regulating membrane fluidity and the cell cycle. Ergosterol biosynthesis pathway is thus a significant target of most existing antifungals, for instance, fluconazole, itraconazole, amphotericin B, terbinafine, *etc* ^23^. In present study, the ergosterol contents of the cells treated with 0.5MIC, MIC and 2MIC of SiW-5 resulted in a dose-dependent reduction of 19.35%, 51.61% and 80.65% *versus* negative control group (Fig. S5, Fig. 3). Furthermore, TEM results evidenced that the membrane of *C. albicans* treated with SiW-5 was indeed damaged, leading to the morphological change from oval form to smaller irregular form, the intracellular content leakage and structural disorganization in the cytoplasm (Fig. 2).

To study the effect of heteropolytungstates on ergosterol biosynthesis, real-time PCR using five of the essential genes involving in ergosterol biosynthesis was conducted. The results revealed a nearly global upregulation including gene *ERG1* (5.95 ± 1.30), *ERG7* (4.90 ± 0.24), *ERG11* (7.40 ± 2.56) and *ERG28* (6.29 ± 0.71), which is consistent with previous reports^24^. When sterol levels are reduced, the expression of ergosterol biosynthesis (*ERG*) genes are substantially increased. This induction can be mediated by Upc2p, which mainly controls the genes for ergosterol synthesis in yeasts. Upc2p can activate the expression of *ERG* genes in response to sterol depletion. The pathway is subject to negative feedback regulation^25,26^.

In the *in vivo* antifungal study, immunosuppressed mice were inoculated with *C. albicans* via tail vein, leading to systemic infections, affecting kidneys, heart, liver, spleen, lung and brain. Because the kidney was the main target organ, histopathological examinations and fungal burdens of kidneys were thus used to evaluate the *in vivo* antifungal efficacy of SiW-5. The groups treated with 10 mg/kg FLC and 100 mg/kg SiW-5 showed 70% and 60% survival (Fig. 4), and obviously reduced the fungal burden (Fig. 5 and Table 2).

In conclusion, a heteropolytungstate (SiW-5) shows potent antifungal activity *in vitro* and *in vivo*.

## Methods

### Materials

RPMI-1640 medium (Sigma) buffered to pH 7.0 with MOPS (Sigma) was used for MIC determination and fungal growth. Potato dextrose agar medium (Beijing Land Bridge Technology Company) was used for fungal growth. Fluconazole was purchased from TCI Company. Ergosterol standard was purchased from Dr. Ehrenstorfer Company. Primescript RT reagent kit (TaKaRa) was used for reverse transcription. SYBR Green I (Roche) was used for Real-time PCR reactions. All reagents were obtained from commercial supplies and used without further purification. The heteropolytungstates, α-1, 2, 3-K_6_H[SiW_9_V_3_O_40_] (SiW-3), K_6_PV_3_W_9_O_40_ (PW-6), α-K_4_PVW_11_O_40_ (PW-8), K_13_[Ce(SiW_11_O_39_)_2_]·17H_2_O (SiW-5), K_13_[Eu(SiW_11_O_39_)_2_]·25H_2_O (SiW-10) were prepared according to the literature^12–16^.

### Fungal strains

29 *Candida albicans*, 8 *Candida glabrata*, 3 *Candida krusei*, 2 *Candida parapsilosis*, 1 *Candida tropicalis*, and 1 *Cryptococcus neoformans* strains were used in this study (Table 1). The strains named after HL were isolated from clinical fungal infection patients in The First Hospital of Jilin University, China and approved by the hospital ethics committee of the First Hospital of Jilin University, and informed consent was obtained from each patient once the purpose and nature of the study had been fully explained. We confirm that all methods were performed in accordance with the relevant guidelines and regulations. The *Candida* species were preliminarily identified according to the colored colony morphology on CHROMagar Candida medium (CHROMagar Co., France) and Vitek 2 automated system (bioMérieux, Marcy l’Etoile, France). All the strains were preserved in the Department of Health Laboratory, School of Public Health, Jilin University, China.

### MIC determination

The MIC assay of heteropolytungstates was carried out following the Clinical and Laboratory Standards Institute (CLSI) document M27-A3 for fungi^27^. Fungal suspensions containing 1 × 10^3^ CFU/mL in RPMI-1640 medium (Sigma-Aldrich Co., USA) buffered to pH 7.0 with MOPS were inoculated into 96-well plates containing serial two-fold dilutions of antifungal drugs. Fluconazole (FLC; TCI Co., Japan) was used as positive control. Final concentrations of heteropolytungstates and FLC were respectively 512-1 *μ*g/mL and 64–0.125 *μ*g/mL. *Candida parapsilosis* ATCC 22019 and *Candida albicans* ATCC 90028 were adopted as quality control strains. The plates were incubated at 35 °C for 24 h (*Candida* spp.) or 48 h (*Cryptococcus neoformans*). A numerical score was given to each well according to the following scale recommended by CLSI (0, optically clear; 1, slightly hazy; 2, prominent decrease inturbidity; 3, slight reduction in turbidity; 4, no reduction in turbidity). The MIC values were defined as the lowest concentrations at which scores of 2 were observed.

### Time kill kinetics

Time-kill curve studies were conducted as described previously^28^. *C. albicans* HL27 were subcultured twice on potato dextrose agar medium (Beijing Land Bridge Technology Company) plates prior to testing. 1 mL of the adjusted fungal suspension (approximately 5 × 10^6^ to 1 × 10^7^ CFU/mL) was added to 9 mL of RPMI-1640 with or without SiW-5. The range of SiW-5 tested was 0.5, 1, 2 times the MIC. The culture was incubated at 35 °C. At predetermined time points (0, 2, 4, 8, 12, 24 h following the addition of SiW-5), a 100 *μ*L aliquot was removed from each culture and serially diluted with sterile normal saline. A 100 *μ*L aliquot was plated onto a potato dextrose agar medium plate for colony count determination. Plates were then incubated at 35 °C for 24 h. Killing of 99.9% (3 logs) of the starting inoculum was defined as a fungicidal effect.

### Transmission electron microscopy

*C. albicans* HL27 were treated with MIC of SiW-5 at 35 °C for 24 h. The cells centrifuged and washed with phosphate buffer solution (PBS) were fixed with 4% glutaradehyde at 4 °C, washed with PBS, post-fixed by OsO_4_ at 4 °C for 2 h and washed with distilled water. After this, the samples were dehydrated with graded ethyl alcoholand acetone, infiltrated with epoxypropane and embedding agent, polymerized at 35 °C for 12 h, 45 °C for 12 h and finally 60 °C for 24 h. Sectioning was done by LEICA EM UC7 Ultramicrotome. After staining with uranyl acetate and lead citrate, the samples were observed under HITACHI H-7650 TEM.

### Assessment of ergosterol content

*C. albicans* HL27 cells were treated with 0.5, 1, and 2 times MIC of SiW-5 and MIC of FLC at 35 °C for 24 h. The cells were centrifuged and washed with PBS. A 0.5 g wet weight of cell mixed with PBS and fresh saponifier was saponified at 80 °C for 1 h and extracted by petroleum ether. Then the extract was volatilized to dryness at 60 °C. The dry residues were dissolved by methanol to 1 mL/g wet weight of cell and preserved at −20 °C. A standard curve of ergosterol standard (Dr. Ehrenstorfer Co., Germany) consists of 0.001, 0.004, 0.015, 0.0625, 0.25, and 1 mg/mL. ergosterol contents were analyzed using LC-20AB prominence Liquid Chromatograph (Shimadzu Co., Japan) including Shimadzu GL C_18_ column (250 mm × 4.6 mm, 5*μ*m). Eluent was methanol/water (97/3, 100% HPLC grade). Flow rate was 1 mL/min. Temperature was 25 °C. SPD-20AV prominence UV/VIS Detector (Shimadzu) was used to detect UV at 282 nm^29^. The ergosterol inhibition ratio = (1 − ergosterol content of treated cells/ergosterol content of untreated cells) × 100%.

### Real-Time PCR

Real-Time PCR was used to measure the transcriptional expressions of the genes involved in ergosterol biosynthesis of *C. albicans* treated with SiW-5. Total RNA was extracted from *C. albicans* HL27 incubated with or without MIC of SiW-5 for 24 h using the hot phenol method as previously described^30^. Reverse transcription was conducted in a total volume of 20 *μ*L with Primescript RT reagent kit (TaKaRa, China). Real-time PCR reactions were performed with SYBR Green I (Roche, China), using qTOWER 2.0 PCR system (Analytic Jena AG, Germany)^31^. The pimer sequences used in Real-Time PCR were listed in Table S2, using 18 S rRNA as the internal control. The expression level of each gene in the SiW-5 treated sample relative to that of untreated sample was calculated using 2^−ΔΔCt^ method.

### Acute toxicity

All animal experiments including acute toxicity and *in vivo* antifungal studies were approved by the Animal Care and Use Committee at Jilin University. All animal procedures were conducted in compliance with the guideline of the China Association of Laboratory Animal Care. The mice were housed at the Laboratory Animal Center, School of Public Health, Jilin University, China. A total of 70 ICR mice (Changchun, China; 20–22 g) were randomly divided into 7 groups, with equal numbers of female and male in each group. SiW-5 was dissolved in sterile water and intragastrically administered to the mice at a single dose of 1300, 1400, 1500, 1600, 1700 and 1800mg/kg body weight, respectively; the control group received sterile water. Then the mortalities were recorded within 14 days. The values of 50% lethal dose (LD_50_) and 95% confidence were calculated by Bliss method.

### *In vivo* antifungal studies

*In vivo* antifungal studies were performed as described previously^32,33^, with some modifications. The *C. albicans* HL27 was used to infect BALB/c mice (Changchun, China; 18–22 g). Immunosuppression was induced in the mice by intraperitoneal injections of cyclophosphamide (CY, 150 mg/kg of body weight) on days-3 and -1, and prolonged by CY injections (150 mg/kg) on days 3, 6, and 9. The immunosuppressed mice were inoculated with 2 × 10^5^ CFU of *C. albicans* in 0.1 mL sterile saline via tail vein on day 0. Treatments consisted of SiW-5 administered at 100, 50 or 25 mg/kg intraperitoneally once a day or FLC administered at 10 mg/kg intraperitoneally once a day 24 h postinfection for 7 consecutive days, containing 14 mice each with equal numbers of female and male. Infection control group was administered sterile distilled water intraperitoneally, containing 10 mice with equal numbers of female and male. Mice were monitored for 15 days after inoculation. Survival rates, periodic acid-Schiff (PAS) staining histopathological examinations and tissue fungal burdens (the number of CFU per gram of kidney) were used to evaluate the *in vivo* antifungal efficacy of SiW-5.

### Statistical analysis

Statistical analysis was conducted using SPSS 13.0. All experiments were performed in triplicate. Data were represented as mean ± SD. Student’s *t*-test was used to test the significance between two groups. One-way analysis of variance (ANOVA) was used to test the significance among different groups. *P* < 0.05 was considered to indicate statistically significant differences.

## Electronic supplementary material

Supplementary Information
