# Supplementary material for: In vitro and in vivo antifungal activities and mechanism of heteropolytungstates against Candida species
Source: Sci Rep. 2017 Dec 5;7:16942. doi: 10.1038/s41598-017-17239-8 (PMC5717275; doi:10.1038/s41598-017-17239-8)
Supplement: Supplementary file 1 — Supplementary Information [file 41598_2017_17239_MOESM1_ESM.doc]

## Supplementary Information

**Title:** *In vitro* and *in vivo* antifungal activities and mechanism of heteropolytungstates against *Candida* species

**Authors:** Han Li, Hongwei Gong, Yanfei Qi, Juan Li, XufengJi, Jiaheng Sun, Rui Tian, Hao Bao, Xiangfu Song, Qiang Chen, Guoliang Liu

**
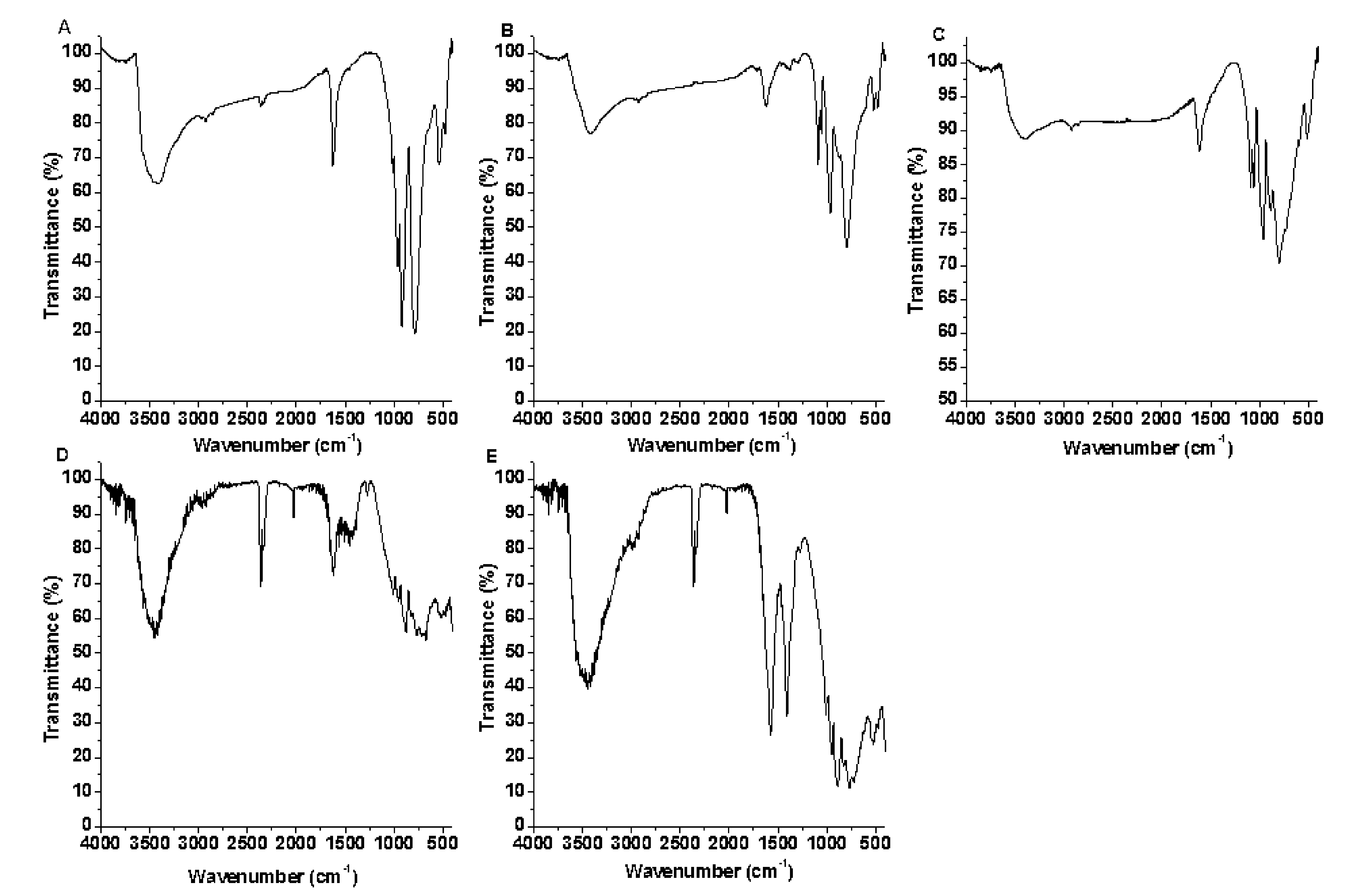
**

**Supplementary Figure S1.** FI-IR spectra of SiW-3(A), PW-6(B), PW-8(C), SiW-5(D) and SiW-10(E).

**Supplementary Table S1.** FI-IR spectraofheteropolytungstates.

|  | ***v*Si/P-O** | ***v*M=O terminal** | ***v*M-O_b_-M** | ***v*M-O_c_-M** | ***v*O-Si/P-O** | ***v*M-O-M** |
| --- | --- | --- | --- | --- | --- | --- |
| SiW-3 | 1007 | 967 | 919 | 785 | 539 | 473 |
| PW-6 | 1089/1059 | 959 | 873 | 794 | 524 | 482 |
| PW-8 | 1092/1056 | 962 | 892 | 797 | 600/510 | - |
| SiW-5 | 1005 | 949 | 876 | 818/774/712 | 523 | - |
| SiW-10 | 1007 | 949 | 896 | 826/769/722 | 522 | 477 |


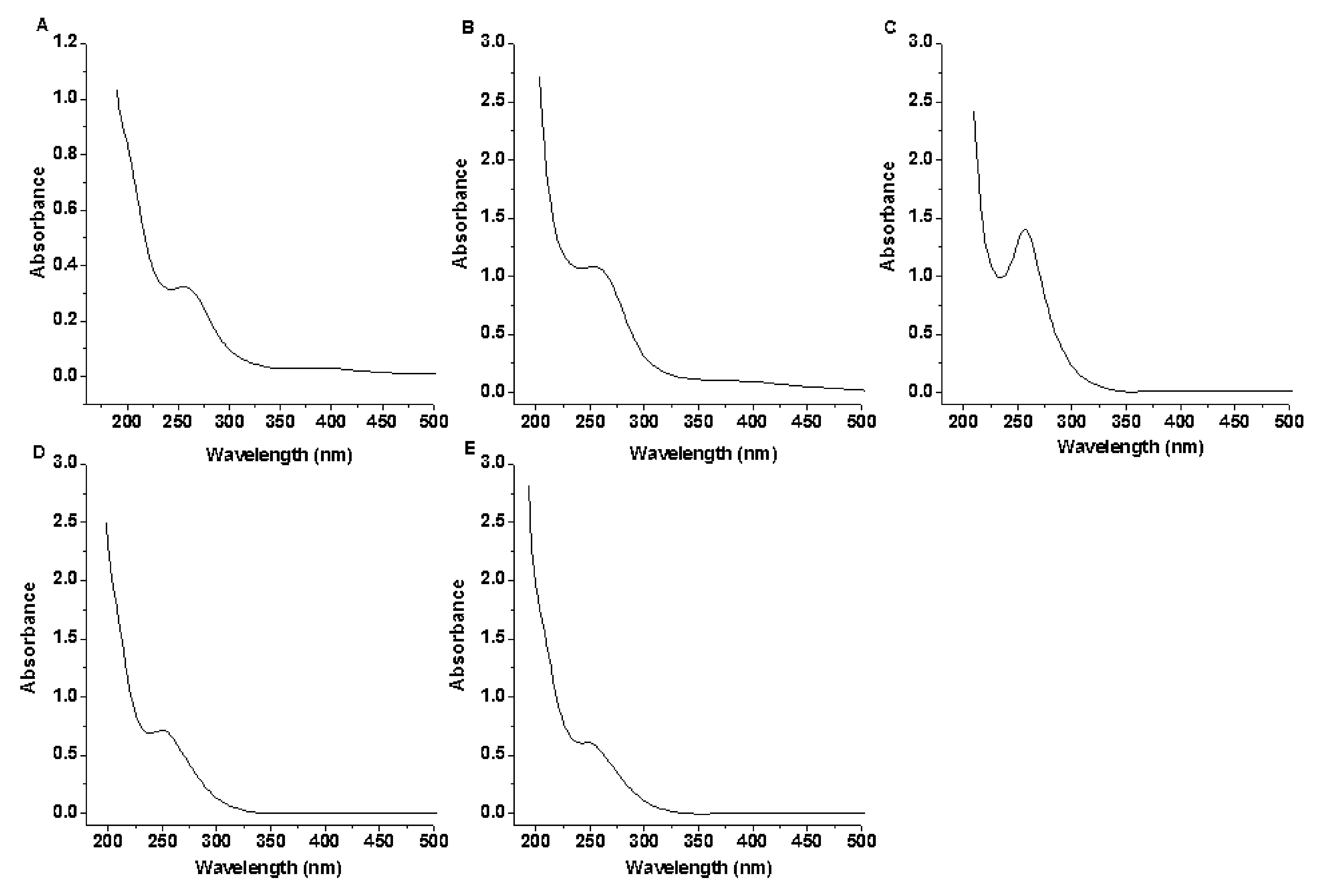


**SupplementaryFigure S2.**UV-Vis spectra of SiW-3(A), PW-6(B), PW-8(C), SiW-5(D) and SiW-10(E).


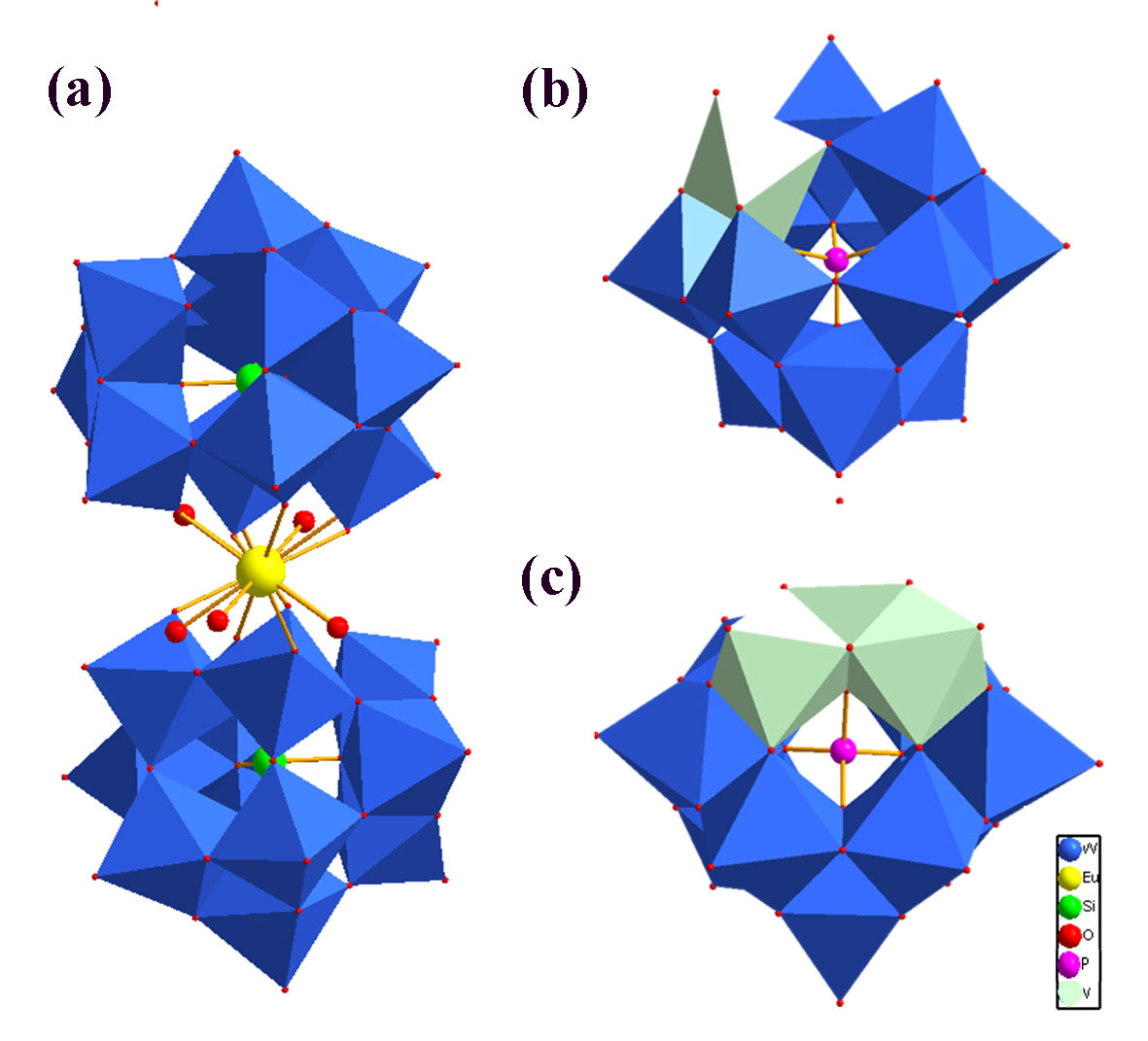


**Supplementary Figure S3.** The representation of structures of heteropolytungstates SiW-10(a), PW-8 (b), PW-6 (c). α-1,2,3-K_6_H[SiW_9_V_3_O_40_] (SiW-3) has the same structure with K_6_PV_3_W_9_O_40_ (PW-6). K_13_[Ce(SiW_11_O_39_)_2_]·17H_2_O (SiW-5) has the same structure with K_13_[Eu(SiW_11_O_39_)2]·25H_2_O (SiW-10). Therefore, only the structures of SiW-10(a), PW-8 (b) and PW-6 (c) were showed.


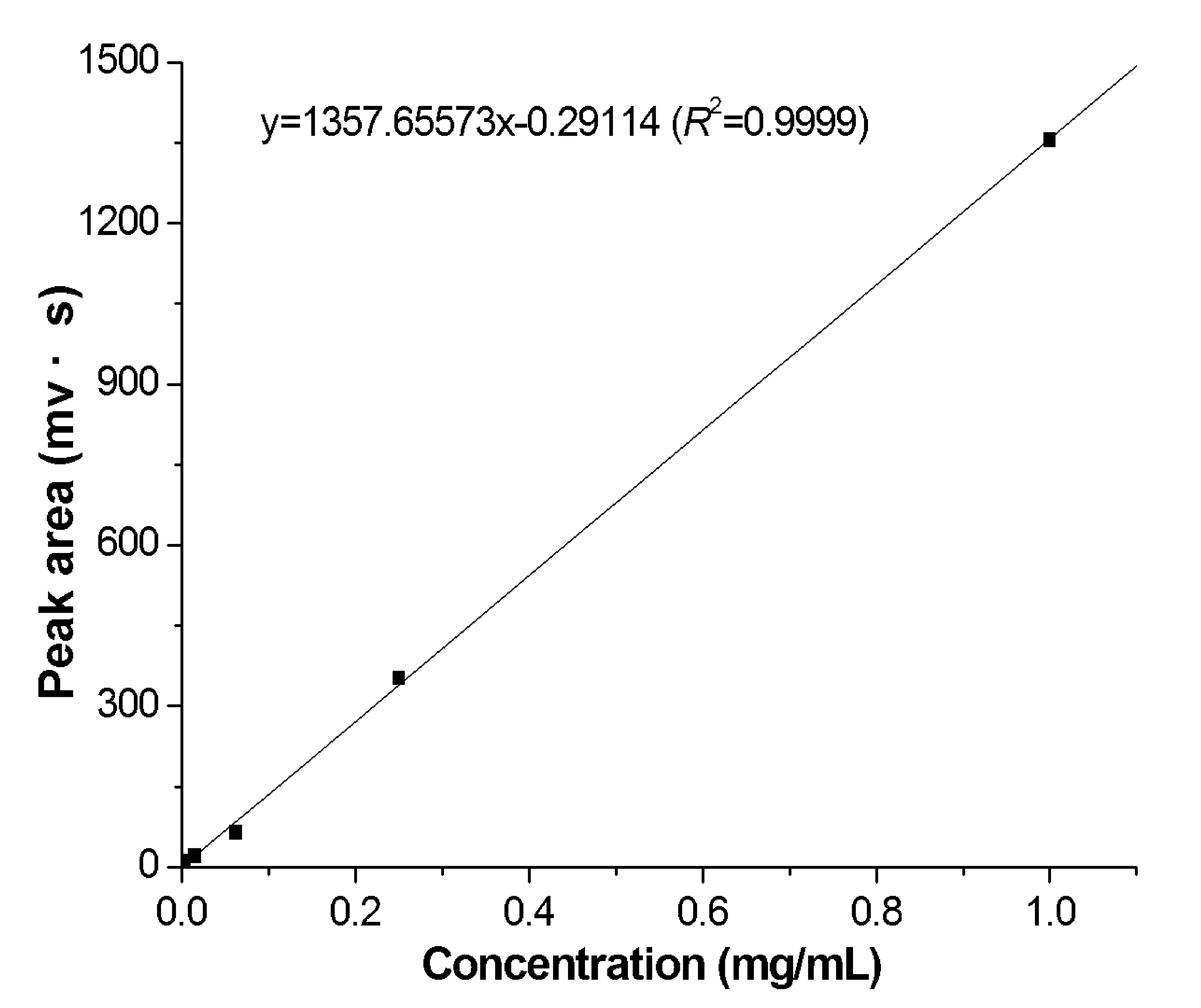


**Supplementary Figure S4.** Standard curve of ergosterol standard included concentrations of 0.001, 0.004, 0.015, 0.0625, 0.25, 1mg/mL.


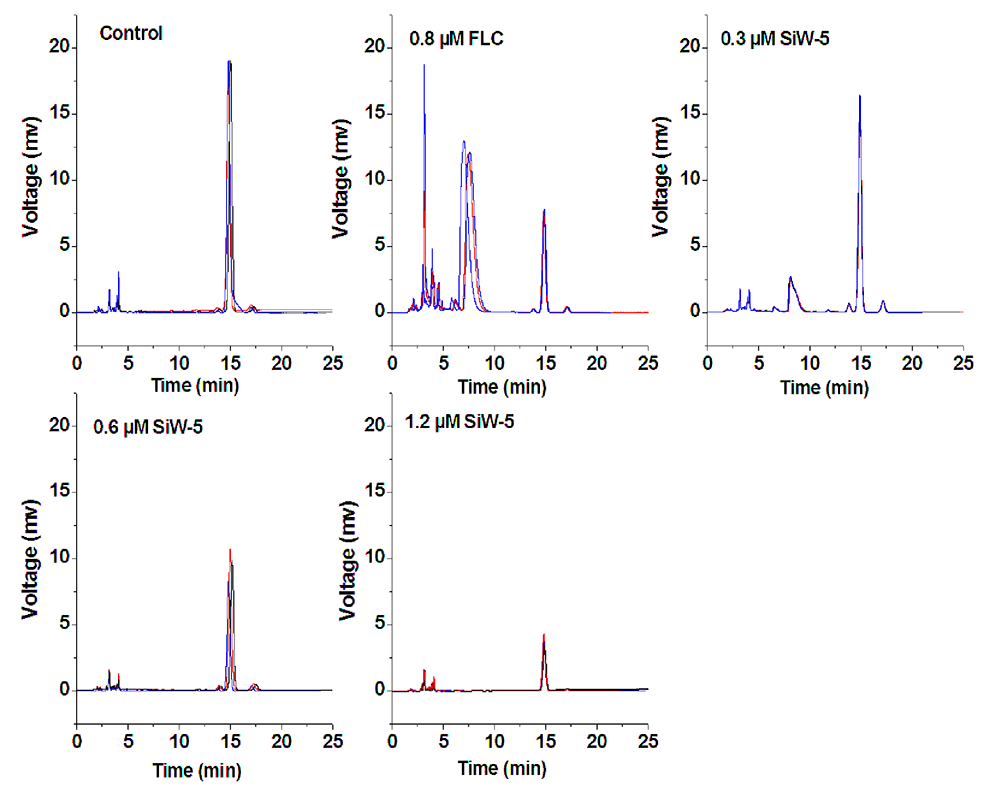


**Supplementary Figure S5.** HPLC graphs of ergosterol in *C. albicans* HL27. The retention time of ergosterol was about 14.798 min. Each graph displayed three repeated experiments.

**Supplementary Table S2.** Primers used for Real-Time PCR.

| **Gene** | **Primer sequence (5’-3’)** | **Size (bp)** |
| --- | --- | --- |
| *18S* | F:TCTTTCTTGATTTTGTGGGTGG | 150 |
|  | R: TCGATAGTCCCTCTAAGAAGTG |  |
| *ERG1* | F: AAGGGCAAAGGTCATGTGTT | 121 |
|  | R: CGTTAGCAGCAGAAGGAGGT |  |
| *ERG7* | F: TTATGCGTCGATGTTTGCAT | 117 |
|  | R: CCACCGTCTGGAAGTTGTTT |  |
| *ERG11* | F: TTTGACCGTTCATTTGCTCA | 110 |
|  | R: GCAGCATCACGTCTCCAATA |  |
| *ERG27* | F: TTGCTGCTGCTTTAGGTCAA | 110 |
|  | R: GTCCAGACCAGTGCTGTCAA |  |
| *ERG28* | F:GCAAGAACTTTTGGAACTTGG | 117 |
|  | R: TGCAGCAATAGCAAATGTGA |  |
